# Dermatofibrosarcoma protuberans – the use of neoadjuvant imatinib for treatment of an uncommon breast malignancy: a case report

**DOI:** 10.1186/s13256-019-2316-0

**Published:** 2019-12-19

**Authors:** Matthew W. McGee, Sarag A. Boukhar, Varun Monga, Ronald Weigel, Sneha D. Phadke

**Affiliations:** University of Iowa Hospitals and Clinics, 200 Hawkins Drive, Iowa City, IA 52242 USA

**Keywords:** Dermatofibrosarcoma protuberans, Neoadjuvant, Imatinib, Breast, Metaplastic breast carcinoma

## Abstract

**Background:**

Dermatofibrosarcoma protuberans is a rare soft tissue malignancy that, if left untreated, can be locally destructive and life-threatening. Dermatofibrosarcoma protuberans is uncommon in the breast, and the similarity of its morphologic features with other spindle cell malignancies can make correct identification difficult. Immunohistochemistry and molecular testing can aid in the correct diagnosis when there is diagnostic uncertainty. Imatinib, a selective tyrosine kinase inhibitor, has been used for adjuvant treatment of dermatofibrosarcoma protuberans following surgical resection. When used as a neoadjuvant treatment, imatinib offers the opportunity to decrease tumor size prior to surgery to lessen the chance for disfigurement.

**Case presentation:**

We present the case of a Caucasian woman who was 46-year-old when she first noted a mass in her right breast in 2015; she was initially diagnosed as having metaplastic breast carcinoma. Mastectomy and systemic chemotherapy were planned; however, after review of pathology at a referral center, the diagnosis was changed to dermatofibrosarcoma protuberans. She was treated with 4 months of neoadjuvant imatinib with adequate tumor shrinkage to perform breast conservation.

**Conclusion:**

This patient’s case stresses the importance of correctly diagnosing this rare breast tumor through the histopathologic appearance of dermatofibrosarcoma protuberans, molecular pathogenesis, and immunohistochemistry. These techniques can help differentiate dermatofibrosarcoma protuberans from metaplastic breast carcinoma and other spindle cell lesions of the breast. This is critical, as the treatment options for metaplastic breast carcinoma significantly differ from treatment options for dermatofibrosarcoma protuberans. This case describes the use of imatinib as a neoadjuvant option to reduce preoperative tumor size and improve surgical outcomes.

## Background

Dermatofibrosarcoma protuberans (DFSP) is a soft tissue malignancy characterized by slow, locally invasive growth [1]. Typically arising in the dermis of the skin, DFSP is usually found on the torso and less commonly on the arms, legs, and neck [1, 2]. DFSP of the breast is rare, and consequently can create a diagnostic challenge [3]. DFSP has a low rate of metastatic spread, however, its local growth can be destructive and disfiguring if left untreated or if treatment is delayed [4]. On clinical examination, DFSP commonly appears as a salmon-colored erythematous plaque, extending into the subcutaneous tissue, fascia, and adjacent muscle with tendril-like growth and microscopic extensions, which can make complete surgical resection difficult [4]. For this reason, targeted therapy aimed at tumor shrinkage may be a valuable preoperative therapy.

DFSP is associated with a distinguishing chromosomal translocation, t(17;22). This results in a rearrangement and fusion of the type I alpha I collagen (*COL1A1*) gene, which is widely expressed in many types of cells, and the beta chain of platelet-derived growth factor (PDGFB) [4]. This molecular aberration leads to the overstimulation of the PDGFB cell surface receptor kinase, subsequently causing tumorigenesis and cellular proliferation [5]. Imatinib is a small molecule tyrosine kinase inhibitor with activity against platelet-derived growth factor receptor (PDGFR) and was approved for the treatment of unresectable and metastatic DFSP in 2006 [4]. Neoadjuvant use of imatinib may be effective in surgically challenging cases or in patients with DFSP tumors that are present in cosmetically sensitive areas [2]. Reduction in tumor burden has been reported when imatinib is used prior to surgery, with up to a 37% reduction in tumor size cited in several studies [2, 5, 6].

While resection is often curative, DFSP diagnosis can be difficult; the potential for misdiagnosis exists due to the similarity in the pathologic appearance of DFSP with other spindle cell tumors [7]. The following case report describes a patient diagnosed as having DFSP of the breast and how the correct diagnosis led to an effective targeted therapy, avoiding the use of cytotoxic chemotherapy.

## Case presentation

**Fig. 1:**
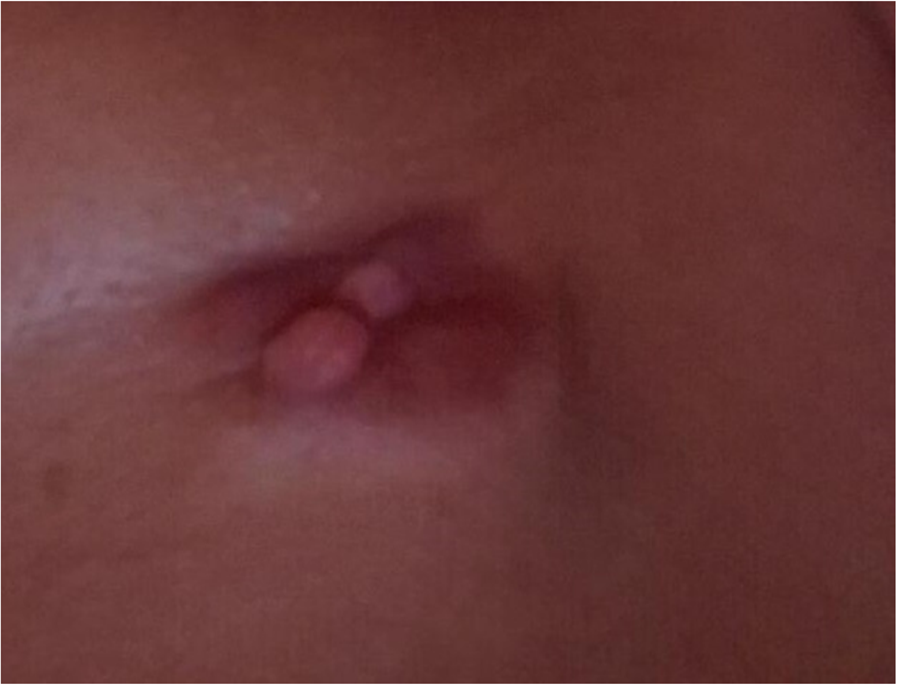
Picture of skin tag over right breast mass in October 2017 that was noticed and photographed by the patient

Our patient was a 46-year-old Caucasian woman when she first noted a mass in her right breast in 2015. A screening mammogram at that time showed scattered fibroglandular densities as well as a skin lesion in the inner right lower quadrant of her right breast, categorized as a BI-RADS 2. No further work up was performed. In October of 2017, our patient noticed a skin tag over her right breast and presented to her primary care provider, who removed it (Fig. 1). She subsequently underwent repeat diagnostic breast imaging with mammogram and ultrasound which showed a bulging 5.2 cm suspicious mass in the right lower quadrant composed of mixed cystic and solid components, classified as BI-RADS 4. She underwent an ultrasound-guided core biopsy of the right breast mass. The pathology specimen was initially read as metaplastic carcinoma of the spindle cell type. The report noted the specimen was negative for estrogen and progesterone receptors as well as the HER2-neu receptor. A computed tomography scan of her chest and abdomen noted no metastatic spread and a right axillary ultrasound showed no lymphadenopathy. She was diagnosed as having clinical T4 N0, stage IIIB metaplastic breast cancer. Neoadjuvant chemotherapy was planned and was to be followed by mastectomy. Prior to this diagnosis, our patient had no significant past medical history. Her family history was significant for breast cancer in her paternal grandmother. Her social history was notable for a 30-pack year smoking history and no significant alcohol use.

**Fig. 2:**
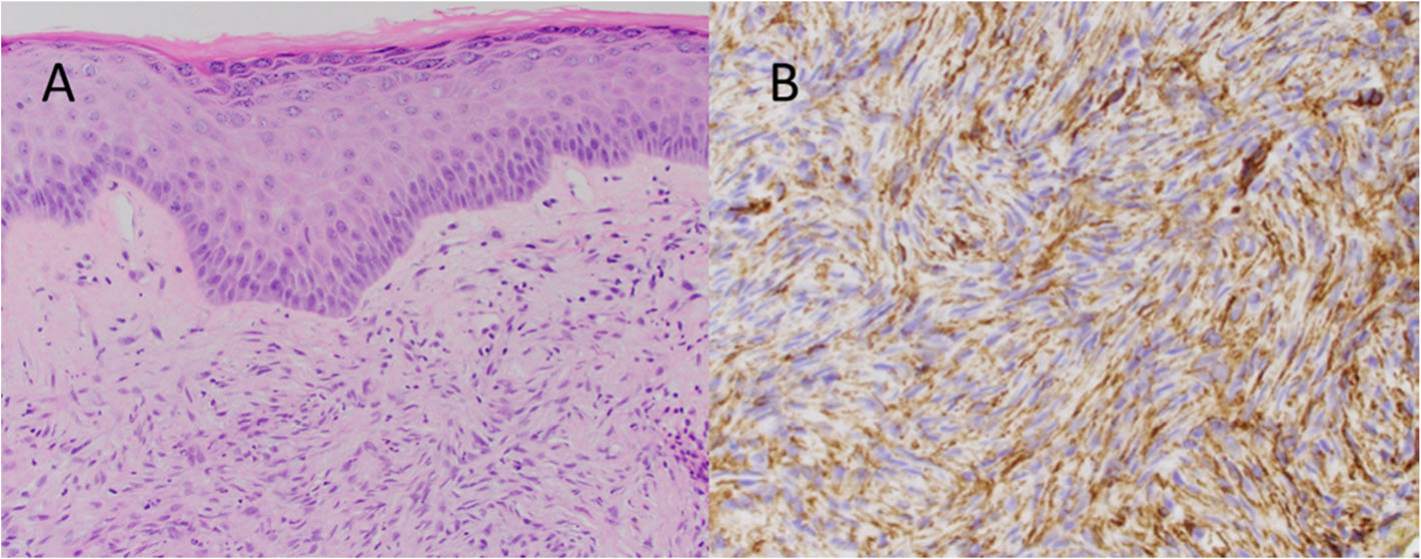
Pathologic microscopy and staining pattern of the biopsy specimen. **a** Section of the right breast mass biopsy showing spindle cell proliferation arising from the interface between the dermis and subcutis and sparing the dermis **b**. Immunostained sections of the biopsy specimen demonstrate uniform positivity of the spindle cells for CD34

**Fig. 3:**
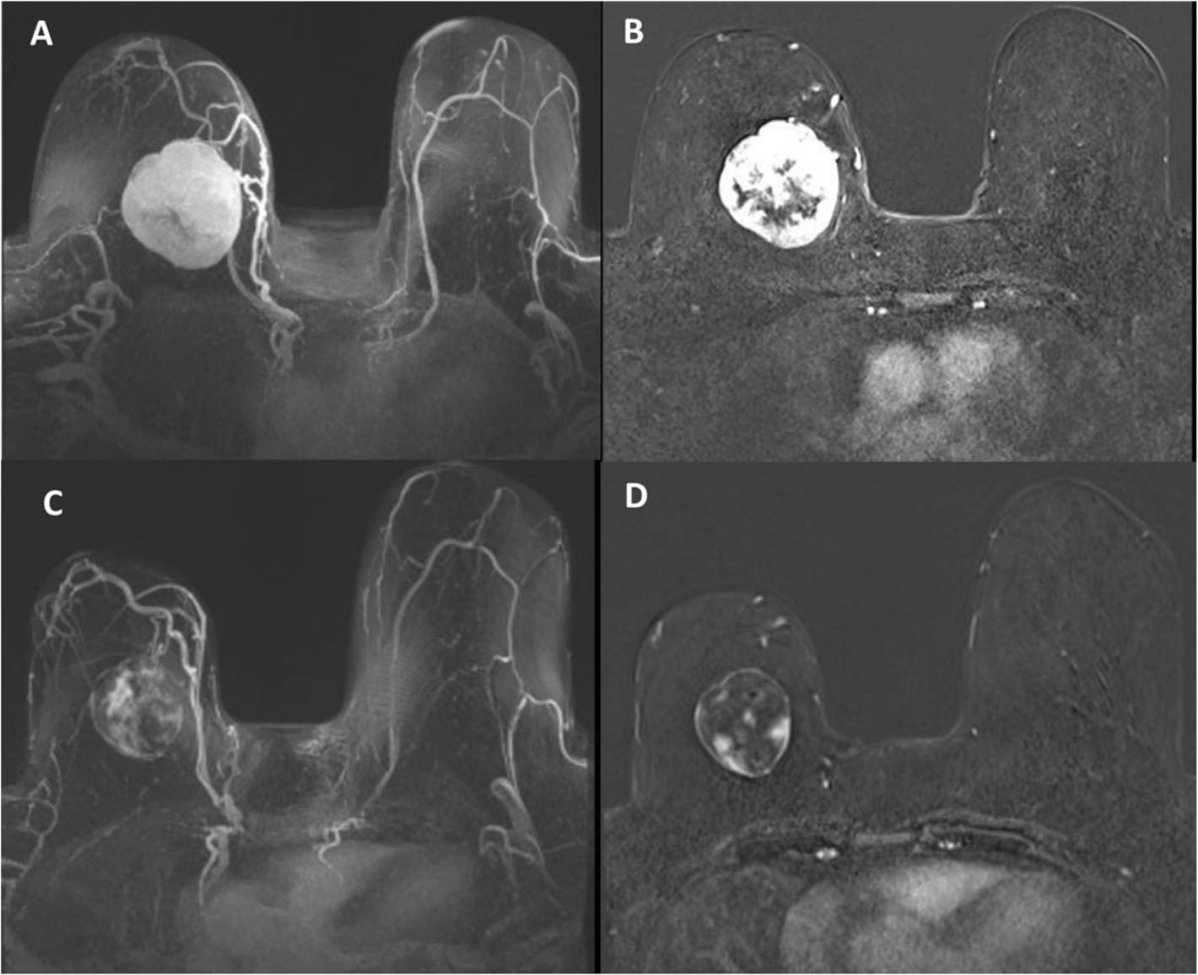
Pre-treatment – axial three-dimensional T1 post-contrast maximum intensity projection (**a**) and post-contrast first phase (**b**) images show a large mass (61 × 64 × 45 mm) with circumscribed margins and heterogenous enhancement in the lower inner region of right breast. Post-treatment – axial three-dimensional T1 post-contrast maximum intensity projection (**c**) and post-contrast first phase (**d**) images show reduction in size (36 × 45 × 38 mm) and greater reduction in enhancement of the mass

**Fig. 4:**
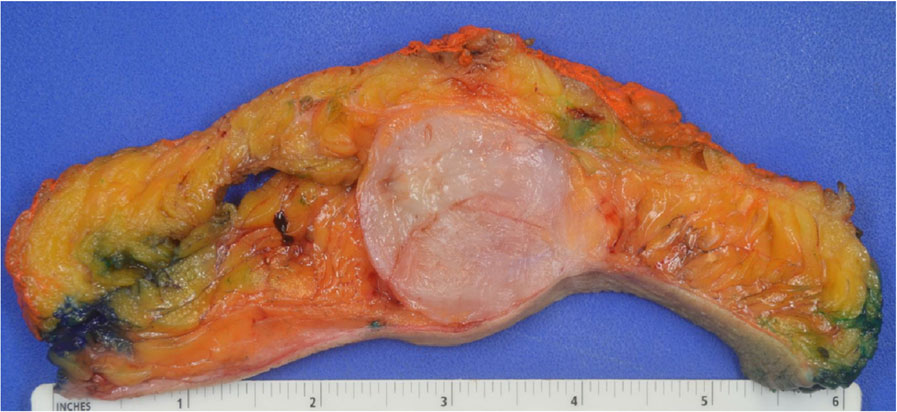
Gross picture of the resection specimen demonstrating skin and subcutaneous tissue with unifocal (up to 5.4 cm) gray-white nodular growth. The mass is unencapsulated but fairly circumscribed, involving mainly the subcutis and focally extending to skin

**Fig. 5:**
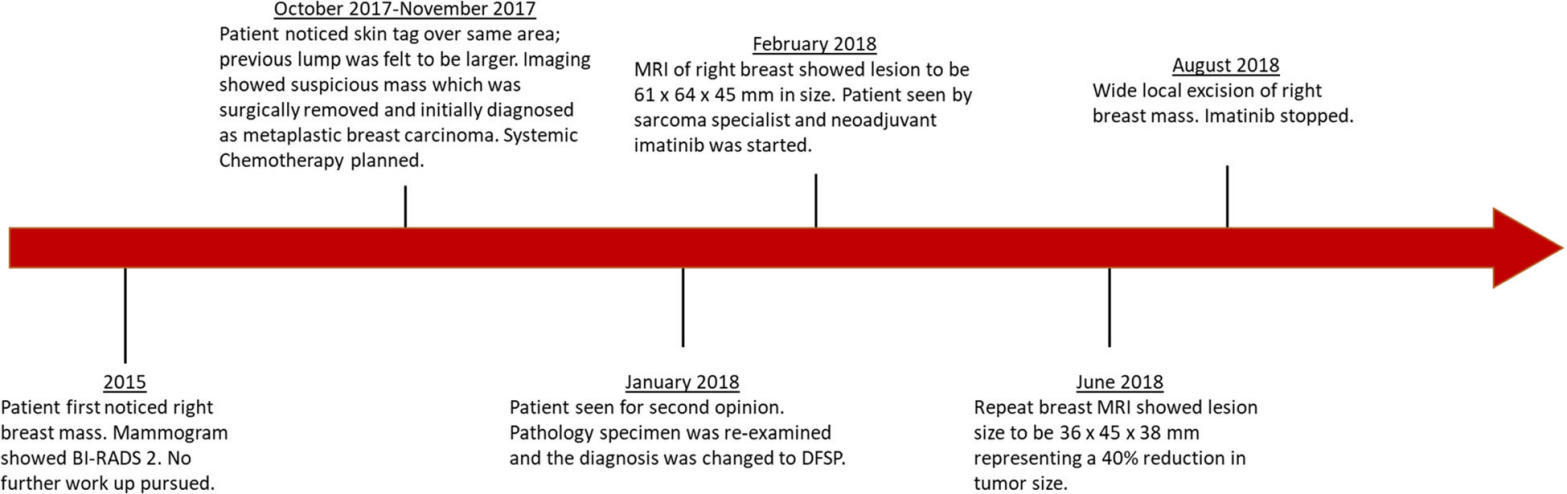
Timeline of the patient’s clinical care. *MRI* magnetic resonance imaging

Due to the rarity of metaplastic breast cancer, a second opinion was sought at our institution in January of 2018. Her physical examination at that time was notable for a 10.5 cm × 10 cm hard mass in the inferior right breast with associated hyperpigmentation and surrounding erythematous skin lesions. On re-examination of the biopsy specimen, the pathology report noted monomorphic spindle cell growth arranged in fascicles with minimal atypia and focal mitotic activity which was observed to arise from the dermis (Fig. 2a). These atypical cells infiltrated into the subcutaneous tissue and focally entrapped fat cells. By immunohistochemistry (IHC), the spindle cells stained diffusely positive for CD34 with focal weak p63 immunoreactivity while negative for CK5, CK7, CK19, CD20, pankeratin, CK5/6, CK903, SMSA, desmin, and S100 (Fig. 2b). Molecular testing revealed the presence of a *COL1A1*-*PDGFB* fusion transcript confirming the diagnosis of DFSP. She was subsequently seen by a breast surgeon and a medical oncologist specializing in sarcoma. Breast magnetic resonance imaging (MRI) measured the lesion to be 61 × 64 × 45 mm. Our patient desired breast-conserving therapy; however, with the initial size of the mass, this was not surgically feasible. She was started on neoadjuvant imatinib at 400 mg daily which she tolerated well with only some minor side effects noted which consisted of fluid retention most noticeable in her face and hands. She was monitored monthly; a repeat breast MRI 4 months after starting imatinib showed a 40% reduction in tumor size (Fig. 3). She underwent wide local excision in August of 2018. Pathology revealed a 5.4 cm DFSP tumor with negative margins and evidence of treatment effect, with 5% tumor necrosis (Fig. 4). No adjuvant therapy was recommended. Throughout the course of her treatment she experienced no other adverse or unexpected events. She continues to follow-up with medical and surgical oncology with annual mammograms and ultrasonography. A timeline of our patient’s clinical course is summarized in Fig. 5.

## Discussion

Spindle cell lesions of the breast can present a conundrum to the pathologist and clinician. Not only do overlapping histological and cytomorphological features make correct diagnosis difficult, but the rarity of mesenchymal tumors also presents challenges [3]. Adding to this difficulty, DFSP represents only 1% of all sarcomas and rarely occurs in the breast, with very few cases having previously been reported [1, 8]. Spindle cell tumors also are exceedingly rare in the breast, comprising 1% of breast malignancies, and are often misdiagnosed, as in this patient [9]. The case presented here demonstrates the importance of histochemical and molecular testing when attempting to differentiate the more atypical neoplasms of the breast. In contrast to metaplastic carcinoma of the breast, which can be treatment-resistant and is associated with poorer outcomes, DFSP is more often a local disease with higher rates of cure [6]. Most importantly, the treatment of DFSP is very different from that of metaplastic carcinoma.

Metaplastic breast carcinoma (MBC) represents a heterogenous group of malignancies with a wide morphological spectrum [7]. The 2011 World Health Organization Working Group classified five distinct subtypes of MBC [10]. The spindle cell subtype of MBC, as was first diagnosed in this patient, often resembles a low-grade sarcoma, making correct identification difficult [11]. On histologic examination, spindle cell metaplastic carcinoma has several architectural patterns with appearances characterized as fascicular, storiform, or haphazard, and with cells described as having infiltrative edges that can obliterate adjacent normal breast tissue rather than grow around them [3, 7]. Furthermore, spindle cell MBC predominantly appears as poorly cohesive sheets of atypical spindle cells that can appear similar to granulation tissue. Spindle cell MBC can also show a variable degree of cellularity, frequent mitotic figures, and can have areas of necrosis and inflammatory infiltrate [7]. The cytological atypia may have a wide variation in appearance from prominent pleomorphism to bland-appearing cells [12].

IHC is an important tool in the diagnosis of MBC [8]. Cytokeratin markers AE1 and AE3, along with vimentin, are usually co-expressed and are the most sensitive markers for this tumor [8]. Expression of p63, along with other cytokeratins, has been used as a marker to identify MBC [13, 14]. The infiltrating spindle cells of MBC may have ducts with prominent myoepithelial cells at the periphery that show diffuse S100 positivity [14]. Spindle cell MBC is typically negative for CD34 [7], but it expresses markers of myoepithelial differentiation such as SMA and S100 in addition to p63, as previously mentioned [12].

DFSP has many histological similarities to spindle cell MBC. DFSP tumors are composed of bland uniform spindle cells and are classically described as being arranged in a storiform or tight whorling pattern [3]. The growth pattern of DFSP is also described as being infiltrative; however, unlike MBC, its infiltrating edges are more subtle with ill-defined borders [15]. The predominant histological characteristic of DFSP is its capacity to invade surrounding tissues and entrap subcutaneous fat lobules [16]. Furthermore, DFSP usually shows little nuclear pleomorphism with low to moderate mitotic activity [17].

IHC is also useful for diagnosing DFSP. In one study of the staining patterns of DFSP, CD34 testing showed a sensitivity and specificity of 94% and 83%, respectively [18]. Tumor cells of DFSP stain negative for factor XIIIa, keratins, and S100 [3]. Our patient’s tumor showed the typical staining pattern of DFSP, with positive staining for CD34 and negative staining for pankeratin, CK5/6, and S100. A further aid for the clinician and pathologist in the diagnosis of DFSP is molecular testing. As previously mentioned, DFSP is associated with the chromosomal translocation t(17;22)(q22;q13)(*COL1A1*;*PDGFB*). With the use of fluorescence *in situ* hybridization studies, which can identify the chromosomal aberration, DFSP can be differentiated from other spindle cell tumors of the breast [19].

Due to its rarity, MBC does not have a standard treatment regimen and clinical practice guidelines for invasive breast adenocarcinoma are often used, including surgery, traditional cytotoxic chemotherapy, and radiotherapy [20, 21]. The preferred treatment for DFSP has historically involved surgical resection with careful margin evaluation [2, 4]. Mohs micrographic surgery may be preferable, owing to its better margin definition and increased tissue-sparing techniques. This technique, however, is not universally performed [22]. Systemic chemotherapy is largely considered ineffective for DFSP [23], and the benefit of radiation therapy is limited, carrying a risk for inducing new or more aggressive tumors [4, 22]. A prior meta-analysis of two retrospective cohort studies revealed no advantage when radiation plus surgery was performed compared to surgery alone [23].

In order to offer our patient breast-conserving surgery, as she desired, neoadjuvant imatinib was recommended and was successful in shrinking the tumor enough to proceed with breast conservation. Studies have looked at response rates of neoadjuvant imatinib with respect to decrease in DFSP tumor size. Kérob *et al.* [5] found a clinical response rate of 36% in a phase II study using 2-month neoadjuvant imatinib therapy. In another study, Han *et al*. [2] reported clinical response rates with an average decrease of 36.9% in preoperative tumor size. In line with this, our patient’s tumor size showed a 40% reduction in tumor size prior to surgical resection. Other case reports have also described the successful use of neoadjuvant imatinib, and these are summarized in Table 1.

**Table 1:** Case reports of neoadjuvant imatinib in the treatment of primary dermatofibrosarcoma protuberans

Study	Disease site	Response	Outcome reported
Fontecilla *et al.*, 2017 [24]	Scalp with extension to periosteum	Partial response	Tumor completely resected
Bekerman *et al.*, 2013 [25]	Scapula	Not reported	Patient developed side effects of hypoxemia and shock requiring intubation. Tumor was resected with positive margins.
Lemm *et al*., 2008 [26]	Scalp	Partial response	No evidence of recurrent disease after surgical resection
Wright and Petersen, 2007 [27]	Scalp	Partial response	No evidence of disease 16 months following resection
Savoia *et al*., 2006 [28]	Anterior chest wall	Partial response	8 months of administration with continued reduction in tumor size
Mehrany *et al*., 2006 [29]	Left cheek	Partial response	18 months following resection patient was disease-free

The strengths of our approach included multidisciplinary management with sarcoma experts leading to the correct diagnosis and, subsequently, the use of a very effective therapy, imatinib. Through the use of neoadjuvant imatinib, our patient was able to achieve her goal of breast conservation. Obtaining a second opinion did result in a slight delay in starting therapy; however, this limitation was overcome by providing a correct diagnosis. Notably, few studies and case reports describe the long-term outcomes of patients treated with neoadjuvant imatinib, therefore continued follow-up and surveillance is needed.

## Conclusion

DFSP of the breast is exceedingly rare, with few cases being previously described. Due to its rarity, DFSP may often be misdiagnosed as a primary breast epithelial malignancy. For this reason, when the diagnosis is unclear or when a sarcoma is suspected, a second opinion from a center with sarcoma experience should be considered. Due to the favorable prognosis of DFSP and its high rate of cure with appropriate therapy, the correct diagnosis of DFSP is imperative. The use of neoadjuvant imatinib has proven to be, both in this case and previous case reports, an effective modality to decrease tumor size and allow for successful excision.

## Data Availability

Data sharing is not applicable to this manuscript as no datasets were generated or analyzed during the current case.

## Abbreviations

*COL1A1*: Type I alpha I collagen
DFSP: Dermatofibrosarcoma protuberans
IHC: Immunohistochemistry
MBC: Metaplastic breast carcinoma
MRI: Magnetic resonance imaging
PDGFB: Platelet-derived growth factor beta
PDGFR: Platelet-derived growth factor receptor
